# ICAM1^+^ gingival fibroblasts modulate periodontal inflammation to mitigate bone loss

**DOI:** 10.3389/fimmu.2024.1484483

**Published:** 2024-11-22

**Authors:** William S. Kim, Kawintip Prasongyuenyong, Annette Ko, Rahul Debnath, Zhaoxu Chen, Jonathan X. Zhou, Emon Shaaf, Kang I. Ko

**Affiliations:** ^1^ Department of Periodontics, School of Dental Medicine, University of Pennsylvania, Philadelphia, PA, United States; ^2^ Department of Oral and Maxillofacial Surgery, Faculty of Dentistry, Prince of Songkla University, Hatyai, Songkhla, Thailand

**Keywords:** stromal cell, innate immunity, periodontitis, CCL2, macrophage, inflammation, host response, fibroblast

## Abstract

Tissue-resident fibroblasts are heterogeneous and provide an endogenous source of cytokines that regulate immunologic events in many osteolytic diseases. Identifying distinct inflammatory fibroblast subsets and conducting mechanistic *in vivo* studies are critical for understanding disease pathogenesis and precision therapeutics, which is poorly explored in periodontitis. Here, we surveyed published single-cell datasets for fibroblast-specific analysis and show that Intercellular Adhesion Molecule-1 (ICAM1) expression selectively defines a fibroblast subset that exhibits an inflammatory transcriptional profile associated with nuclear factor-κB (NF-κB) pathway. ICAM1^+^ fibroblasts expand in both human periodontitis and murine ligature-induced periodontitis model, which have upregulated expression of CCL2 and CXCL1 compared to other fibroblast populations. Using a mouse model to selectively target gingival stromal cells, we further show that disruption of an inflammatory pathway by inhibiting transcriptional activity of NF-κB in these cells accelerated periodontal bone loss. Mechanistically, this was linked to a reduction of CCL2 expression by the ICAM1^+^ fibroblasts, leading to impaired macrophage recruitment and efferocytosis that was associated with persistent neutrophilic inflammation. These results may have a significant therapeutic implication as ICAM1^+^ gingival fibroblasts exert a protective response by regulating innate immune responses that are needed for the controlled inflammatory events in early stages of periodontitis.

## Introduction

Periodontitis is a prevalent oral inflammatory disease characterized by the progressive destruction of the supporting periodontal apparatus, which ultimately leads to tooth loss. A prevailing pathologic mechanism responsible for exacerbated periodontal bone loss is the aberrant inflammation that favors osteoclastogenesis (1). Studies have demonstrated that both innate and adaptive arms of immunity are altered in periodontitis. For example, heightened neutrophilic inflammation in acute and chronic phases of periodontitis causes connective tissue damage by releasing destructive proteolytic enzymes (2). Moreover, T-helper cells and γδT cells that secrete a pro-inflammatory cytokine interleukin-17 are overly active and decouple bone metabolism to mediate alveolar bone loss (3, 4). The hyperactivated immune response is largely absent in steady-state conditions, suggesting that endogenous resident cells may be important initiators for downstream events that lead to osteolytic inflammation in periodontitis. Non-immune tissue resident cells, i.e. mesenchymal cells, have been shown to drive the pathogenesis of other inflammatory diseases such as rheumatoid arthritis and intestinal colitis (5, 6). However, relatively little is known about the functional significance of mesenchymal sentinel cells in periodontal pathogenesis.

Gingival fibroblasts are abundant stromal cells that maintain the structural integrity of the lamina propria within tooth-associated gingiva. Recent advances in single-cell research have highlighted that fibroblasts are comprised of heterogenous populations with distinct molecular signatures (7). In rheumatoid arthritis, which shares many pathologic features with periodontitis (8), a pro-inflammatory fibroblast subset mediates abnormal recruitment of leukocytes that induces destruction of the synovium, which was proven *in vivo* (9). In periodontal literature, emerging single-cell RNA-seq (scRNA-seq) studies unanimously identify a gingival fibroblast subset that uniquely expresses cytokine and chemokine genes responsible for trafficking both innate and adaptive immune cells (10, 11). However, *in vivo* mechanistic studies to determine the functional significance of these fibroblasts are surprisingly lacking. Furthermore, most studies on gingival fibroblasts rely on *in vitro* culture systems, which may not capture complex cellular interactions within the diseased periodontal niche (12, 13). This is particularly important to understand as controlled inflammation is necessary for the antibacterial immune response but can also be destructive when persistently present in the periodontium (14).

A major challenge in studying inflammatory fibroblast population is the lack of a definitive surface marker for cell isolation and limited *in vivo* mouse models that selectively target fibroblast lineage cells. In skin fibrosis, *engrailed-1* lineage cells identify scar-forming fibroblasts, inhibition of which can minimize scar formation (15). In a rheumatoid arthritis model, specific deletion of fibroblast activation protein-α-expressing fibroblasts mitigates synovial inflammation and joint destruction (9). In contrast, we recently reported that lineage cells labeled by the promoter activity of paired related homeobox-1 (Prx1) label immunomodulatory fibroblasts needed for proper cutaneous and oral wound healing to occur (16). Thus, whether inflammatory fibroblasts are protective or destructive may be disease-specific and context-dependent, although a potential role of these fibroblasts in periodontal disease pathogenesis remains unclear.

Here, we combine the survey of published scRNA-seq data, validation assays using human gingival tissues, and a murine experimental periodontitis model to identify ICAM1 (Intercellular Adhesion Molecule 1) to be a cell surface marker that distinguishes inflammatory fibroblasts. *In vivo* studies to disrupt inflammatory transcriptional activity of the canonical NF-κB pathway in fibroblasts demonstrate that these cells are needed to prevent excessive periodontal bone loss. Mechanistically, this was linked to an insufficient production of CCL2 and perturbed modulation of macrophage recruitment and efferocytosis that control excessive neutrophilic damage to periodontium. Together, our study highlights a protective function of inflammatory gingival fibroblasts that mount an appropriate innate immune response in periodontitis to minimize tissue damage. Moreover, it implicates the use of an ICAM1 antibody-based cell sorting method as an effective strategy to study inflammatory gingival fibroblasts.

## Materials and methods

### Human tissues

Discarded gingival tissues were collected from patients between the ages of 18-70 undergoing resective surgery at the University of Pennsylvania School of Dental Medicine. Marginal gingiva from the clinical health group was derived from patients undergoing crown lengthening or tooth extraction where periodontal probing depth was ≤3mm. Marginal gingiva from the periodontitis group was from patients undergoing osseous surgery, where periodontal probing depth was ≥5mm with a designated periodontitis stage II or III diagnosis according ref (17). Exclusion criteria included history of diabetes and smoking. The collection and use of discarded biospecimen were approved under IRB #844933 at the University of Pennsylvania, and informed consent was obtained from all participants.

### *In vivo* animal model

Animal studies were carried out in compliance with IACUC-approved protocols (#804855 and #807062). Mice were purchased from the Jackson Laboratory as follows: C57BL/6J (B6, #000664), B6.Cg-Tg(Prrx1-cre)1Cjt (Prx1Cre, #005584), B6.Cg-Gt(ROSA)26Sort9(CAG-tdTomato)Hze/J (R26R^tdTomato^, #007909), and B6.Cg-Cl2tm1.Pame/J (Ccl2^mCherry^, #016849). Ikbkb^flox/flox^ was provided by Dr. Michael Karin (UC San Diego). Control mice were obtained from the same litter unless specified otherwise. All animal experiments were initiated at 6-10-week-old of age. Mice were anesthetized with a 2-4% ketamine solution during surgical procedures and prior to euthanizing via intraperitoneal injection. Mice were euthanized after anesthetization using a cervical dislocation method.

### Ligature-induced periodontitis

Mice were anesthetized, and a 6-0 silk ligature was tied around the second maxillary molar as previously described (18). Mice were euthanized at indicated end timepoints, and maxillary tissues were harvested and fixed for downstream analyses including micro-CT analysis, histomorphometric analysis, immunofluorescence, and TRAP staining.

### Flow cytometry and sorting

Human tissues were isolated and processed within 30 minutes of dissection. Gingival tissues were minced and digested using 0.15 mg/ml DNase I, 3.2 mg/ml CollagenaseIV and 2.6 mg/ml Dispase II for 1h at 37C under constant agitation. After straining through 70um filter, red blood cells were lysed in ACK buffer, nonspecific binding blocked with antibody against CD16/32, and stained with fluorophore-conjugated antibodies and viability dye. Data was acquired using LSRII (BD Biosciences) and analyzed using FlowJo software (10.8.0). Fluorescence-minus-one (FMO) controls were used to determine gating strategy. In other experiments, fluorescence-based sorting was performed on FACSAria (BD Biosciences), and ~10^4^ cells were separated directly into lysis-buffer containing tubes prior to mRNA extraction. For murine studies, ligatured teeth were extracted after euthanasia, and gingival collar ~2mm in width was dissected circumferentially by blunt dissection along the osseous floor of maxilla. Tissue digestion for single-cell preparation, staining, and analyses were carried out as described above. The list of antibodies is available in **Appendix Table 1**.

### Computational analysis

Previously published scRNA-seq datasets (GSE152042, GSE164241, and GSE217720) were downloaded and pooled for integrative analysis. Downstream analyses were performed using R language, and data visualization was performed using Seurat (v.3) (19). Cells that expressed <200 or >5,000 UMI or more than 15% mitochondrial gene expression were excluded. Datasets were integrated via Seurat Integration (Seurat 3). Mutual nearest neighbors (anchors) were found using the SelectIntegrationFeatures and FindIntegrationAnchors functions. The data was integrated using the IntegrateData function with a k.weight of 90. Dimensional plots of the integrated dataset were split by individual sample to evaluate adequate cell type mixing. Additionally, integration was quantitatively analyzed using the local inverse Simpson’s Index (LISI) (20). Fibroblasts were selected based on the enriched expression of *COL1A2, COL3A1*, *DCN, FAP* and *PDGFRA*. Differentially expressed genes were calculated using Findmarker function with default Wilcoxon rank-sum analysis. Gene module score for inflammatory gene set was determined by creating a list of known CCL- and CXCL- genes. Gene expression ratio score was calculated by multiplying average log2(fold-change) and the ratio between gene expression percentage in cluster 0 versus other fibroblast clusters. Signaling pathways of fibroblast subclusters were analyzed by via PROGENy (21).

### Micro-CT analysis

Murine maxillae were fixed in a solution of 10% formalin before being washed with distilled water twice and placed in a solution of 1x PBS for uCT imaging. Maxillae were imaged with a Scanco µCT 45 (Scanco Medical AG, Switzerland) at an applied voltage of 55 kVp and a voxel size of 14.6 µm. Images were reconstructed and analyzed with Dragonfly (Windows, Ver. 2022.1.0.1259). µCT sections were used to evaluate bone resorption with a distance and area measurement. Teeth were positioned in the sagittal plane where the apex of all roots was visible, and the dental pulp of all teeth extended to the apex of all buccal roots. CEJ-ABC distance was quantified by measuring vertical distance between CEJ-CEJ to the most coronal aspect of alveolar bone between first and second maxillary molar teeth. For bone area remaining, an arbitrary 700µm area below CEJ-CEJ to the base of the alveolar bone perpendicular to occlusal plane was used to quantify total periodontium area and radiopaque bone remaining percent.

### Histomorphometric analysis

Murine tissue samples were fixed in a 10% formalin solution immediately after extraction and incubated at 4 C for 18-24 hours. Samples containing bones or teeth were then decalcified in a solution of 14% EDTA (pH 7.4) for 2 weeks with solution changes every three days. Samples were then dehydrated and embedded in paraffin using standard protocols. Samples were then cut at 5um, rehydrated, and stained with hematoxylin and eosin. To measure bone loss, a line (CEJ-CEJ line) was drawn connecting the cemento-enamel junctions of the first and second molars. Another line was drawn starting from the midpoint of this line down to the alveolar bone crest and was used to measure cementoenamel junction to alveolar bone crest distance (CEJ-ABC distance).

### Immunofluorescence

Frozen or paraffin-processed samples were used for immunofluorescence experiments. Briefly, cryosections were cut at 10um and rehydrated in 1X PBS with 0.05% Tween-20, whereas paraffin sections at 5um were deparaffinized, hydrated prior to non-specific blocking in buffer containing 1% BSA, 0.1% Triton-X, 0.05% Tween-20, and 1% serum matching the host species of the secondary antibody. Immunofluorescence staining was performed using primary and secondary antibodies using standard protocols, and detailed antibody information is provided in **Appendix Table 1**.

### TRAP staining

Paraffin-processed sections were rehydrated and stained with a TRAP staining solution (pH = 4.85) for osteoclasts before being washed and counterstained with a solution of 0.08% fast green (CAS: 2353-45-9). TRAP staining solution consisted of 184 mg/ml sodium acetate anhydrous (CAS: 127-09-3), 284 mg/ml L-(+) tartaric acid (CAS: 6106-24-7), 5.6% glacial acetic acid (CAS: 64-19-7), 0.1 mg/ml naphthol (CAS: 1596-56-1), 0.5% ethylene glycol monoethyl ether (CAS: 110-80-5), and 0.6 mg/ml fast red violet LB salt (CAS: 32348-81-5). PH adjustment was made with the addition of sodium hydroxide (Carolina Biological Supply, CAS: 1310-73-2) or glacial acetic acid. All chemicals were from Sigma-Aldrich.

### Image analysis

Images of histological and immunofluorescence slides were taken using the Keyence BZ-X800 microscope and analyzed using the associated BZ-X800 Analyzer software. Image analysis was conducted using ImageJ or QuPath software (22) to count positive and double-positive cells in a given area by at least two examiners in a blinded manner.

### Quantitative PCR

Cells were directly sorted into cold lysis buffer. RNA content was extracted using RNeasy Micro Kit (Qiagen) using the manufacturer’s protocols. The High-capacity CDNA synthesis kit (Applied Biosystems) was used to generate cDNA. Fast power SYBR Green (Applied Biosystems) and the Step-One Plus machine (Thermo Fisher Scientific) were used to perform qPCR. To normalize the data, L32 was used as a housekeeping gene. List of primers can be found in **Appendix Table 2**.

### *In vitro* immunocytochemistry

8-week-old B6 mice were used to harvest palatal gingiva and digested into a single-cell suspension. Primary oral fibroblasts were plated on a 96-well plate containing DMEM (Gibco, #11885-084) supplemented with 10% FBS and 1X antibiotic-antimycotic and incubated at 37°C in a humidified atmosphere with 5% CO_2_ until 90% confluency. Fibroblasts were stimulated with a combination of TNF (10 ng/ml) (R&D: 410-MT-025/CF) and lipopolysaccharide from *P. gingivalis* (LPS-Pg) (1 ug/ml) (Sigma-Aldrich: SMB00610) for 24h. Cells were fixed, blocked in a buffer containing 1% BSA, 0.1% Triton-X, 0.05% and Tween-20, and incubated with primary antibodies against ICAM1 at 4°C overnight. After incubation with the secondary antibodies, cells were washed and stained with DAPI for 5 min. The immunoreactive proteins were then visualized under the fluorescent microscope.

### *In vitro* phagocytosis assay

Bone marrow-derived macrophages (BMMs) were generated following a published protocol (23) using macrophage colony-stimulating factor (20 ng/ml, PeproTech: 315-02) for 5 days. BMMs were then stimulated with low-dose LPS (10 ng/ml) for 24h to induce macrophage phagocytotic phenotype, followed by incubation in conditioned media from the ICAM1^+^ enriched or control fibroblasts, with or without neutralizing anti-CCL2 monoclonal antibody (20 µg/ml, eBioscience, 16-7096-81). For the phagocytosis assay, latex beads (2 µm diameter, carboxylate-modified polystyrene, fluorescent red; L3030, Sigma) were added to the BMMs at a 10:1 ratio (beads to BMMs) and incubated for 3 hours at 37°C. BMMs were then collected, washed with PBS to remove non-phagocytosed beads, and analyzed by flow cytometry.

### *In vitro* treatment with NF-κB inhibitor

Primary oral fibroblasts were generated and pre-stimulated with or without a combination of LPS and TNF as described above. Cells were washed and incubated in media containing selective NF-κB inhibitor (BMS-345541, 10uM, Cayman Chemical) for 6h. Cells were collected for flow cytometry analysis, and supernatant was used for ELISA assay using mouse MCP-1 ELISA kit (Biolegend, 446207) according to the manufacturer’s instructions.

### Statistical analysis

Statistical analysis was conducted on Graphpad Prism software and Rstudio. All data represent mean ± SEM. Normal distributions were verified with QQplots and homogenous variances were verified using Bartlett’s test for homogenous variances. Student’s t-test or Welch’s t-test were used for comparing two groups to account non-homogenous variances. Mann-Whitney U test was used for nonparametric analyses. One-way ANOVA and Šidák’s correction and Brown Forsythe AVOVA with Dunnett’s T3 Multiple comparisons were used for pairwise comparisons. Statistical significance was determined with a p-value < 0.05. Experiments were independently replicated at least two times, and each data point represents an individual animal unless specified otherwise.

## Results

### Identification of ICAM1^+^ fibroblast subset with inflammatory features in human periodontitis

**Figure 1:**
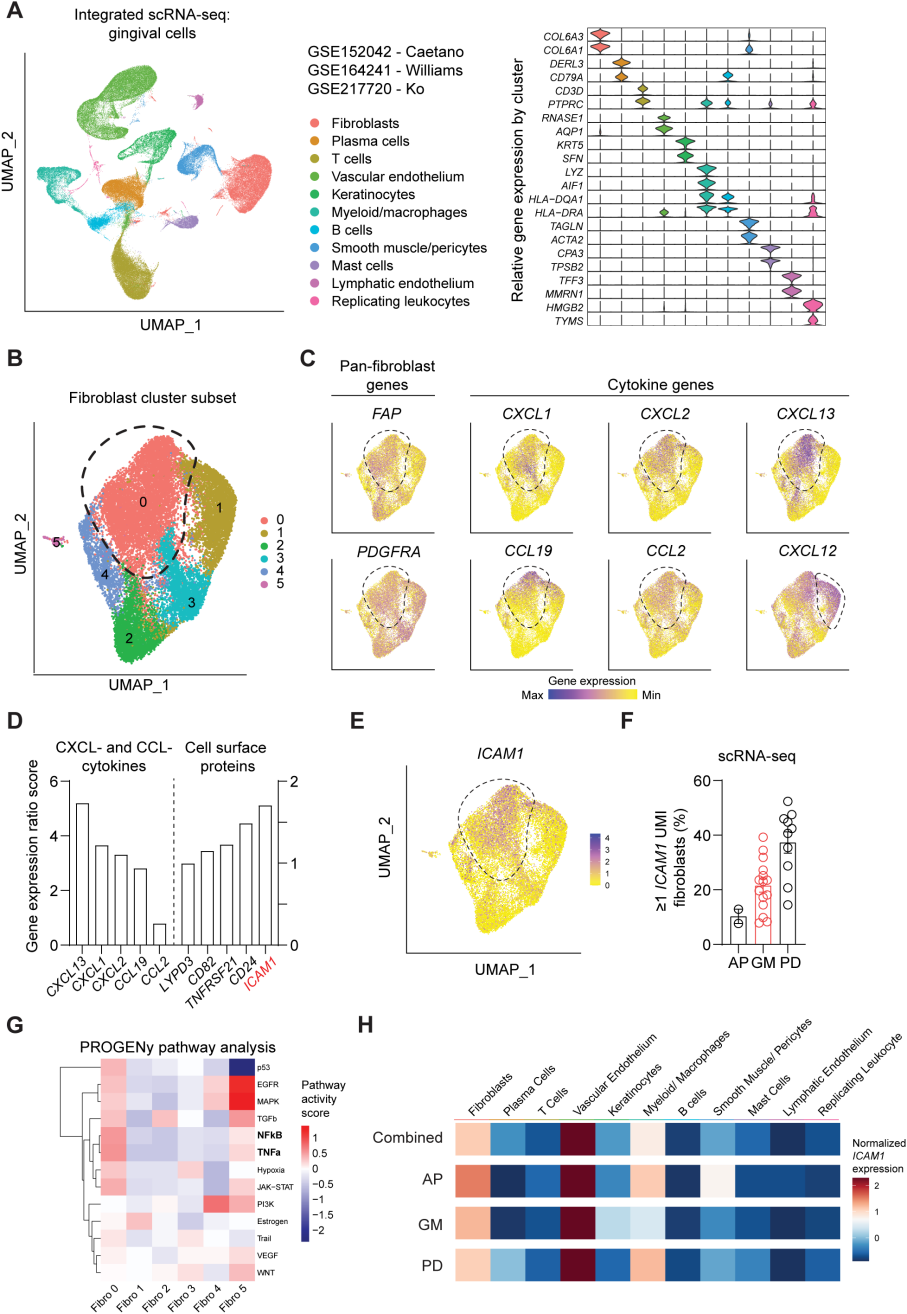
Single-cell RNA-seq analysis of public oral tissue atlas identifies ICAM1^+^ inflammatory fibroblasts. **(A)** Left, uniform manifold approximation and projection (UMAP) plot of n = 27 patients with 113,154 single-cells from integrated data set from ref (10, 11, 24), colored by major cell types. Right, stacked violin plot illustrating characteristic marker gene expression across each identified cell population in the integrated dataset. **(B)** UMAP plot of subset gingival fibroblast clusters from integrated public scRNA-seq dataset. **(C)** UMAP of feature plots for pan-fibroblast marker *PDGFRA* and *FAP* and fibroblast-derived *CCL-* and *CXCL-* cytokine genes. Dashed lines demarcate cluster 0, except *CXCL12* plot that designates fibroblast cluster 1. **(D)** Gene expression ratio score of significantly expressed CXCL- and CCL- cytokine genes and cell surface protein genes in cluster 0. **(E)** UMAP of feature plot for *ICAM1* expression across the fibroblast clusters. Dashed line demarcates cluster 0. **(F)** Quantification of fibroblasts that express ≥1 unique molecular identifier (UMI) for *ICAM1*. Each dot represents the percentage value of an individual patient. AP, anterior palate; GM, gingival margin (healthy); and PD, periodontitis. **(G)** Heatmap illustrating the activity of pathway-responsive genes (PROGENy) across different fibroblast clusters. **(H)** Heatmap illustrating *ICAM1* expression across cell populations in datasets that combine all oral tissues, AP, GM or PD. Scale represents centered log-ratio normalized *ICAM1* expression for each condition.

We first sought to identify a cell surface marker that labels an oral fibroblast subpopulation with inflammatory signatures. We took advantage of publicly available human scRNA-seq atlas datasets and analyzed oral fibroblast subsets that are found in different locations in the oral cavity such as tooth-associated gingiva from healthy group (gingival margin, GM), from those diagnosed with periodontitis (PD), and non-tooth associated gingiva from the hard palate (anterior palate, AP) (10, 11, 24). The data was pre-processed to remove dead cells with >15% mitochondrial gene expression and doublets with high gene feature counts (**Supplementary Figures 1A, B**). Dimensional plots juxtaposing the unintegrated and integrated data showed a drastic improvement in mixing across sample and batch groups, and each patient dataset showed sufficient mixing at an individual level (**Supplementary Figure 1C**). Furthermore, the LISI for unintegrated and integrated were also calculated to quantitatively evaluate batch effects. The integrated data had a median LISI approximately 3-fold greater than the unintegrated data, with an unintegrated median LISI of 2.27 (95% CI [2.25,2.28]) to an integrated median LISI of 6.10 (95% CI [6.09,6.12]) (**Supplementary Figure 1D**). The integrated atlas was then processed to designate cell types based on enriched expression of canonical genes for each cluster, which generated 11 clusters (**Figure 1A**). To focus our analysis on oral fibroblasts, we generated subset clusters that expressed fibroblast genes and examined it at a higher granularity to identify those that exhibited elevated cytokine signatures. Among 6 fibroblast subgroups, cluster 0 was the most abundant and highly expressed cytokines such as *CXCL13, CXCL1, CXCL2, CCL19* and *CCL2* but not *CXCL12* which was also expressed in cluster 1 (**Figures 1B, C**). Given this enriched expression of CCL- and CXCL- cytokines, we further analyzed this inflammatory fibroblast cluster by calculating gene ratio scores, such that the significantly upregulated genes that are highly expressed in cluster 0 but not in other clusters are assigned with top scores. We selectively examined differentially expressed genes that encode for surface proteins and identified *ICAM1* (encoding for CD54) to be upregulated in this inflammatory fibroblast cluster (**Figures 1D, E**). When the scRNA-seq dataset was further stratified by individual patients, *ICAM1^+^* fibroblast percentage was the highest at approximately at 40% in those diagnosed with periodontitis when compared to those with healthy marginal gingiva or palatal gingiva (**Figure 1F**). PROGENy pathway analysis further confirmed that *ICAM1^+^* enriched cluster 0 was highly implicated in active inflammatory pathways associated with TNF and NF-κB signaling pathways (**Figure 1G**). However, *ICAM1* gene expression was upregulated in non-fibroblast clusters such as endothelial cells and macrophages (**Figure 1H**), indicating the need for combinatorial inclusion of pan-fibroblast specific markers to isolate these inflammatory gingival fibroblasts.

**Figure 2:**
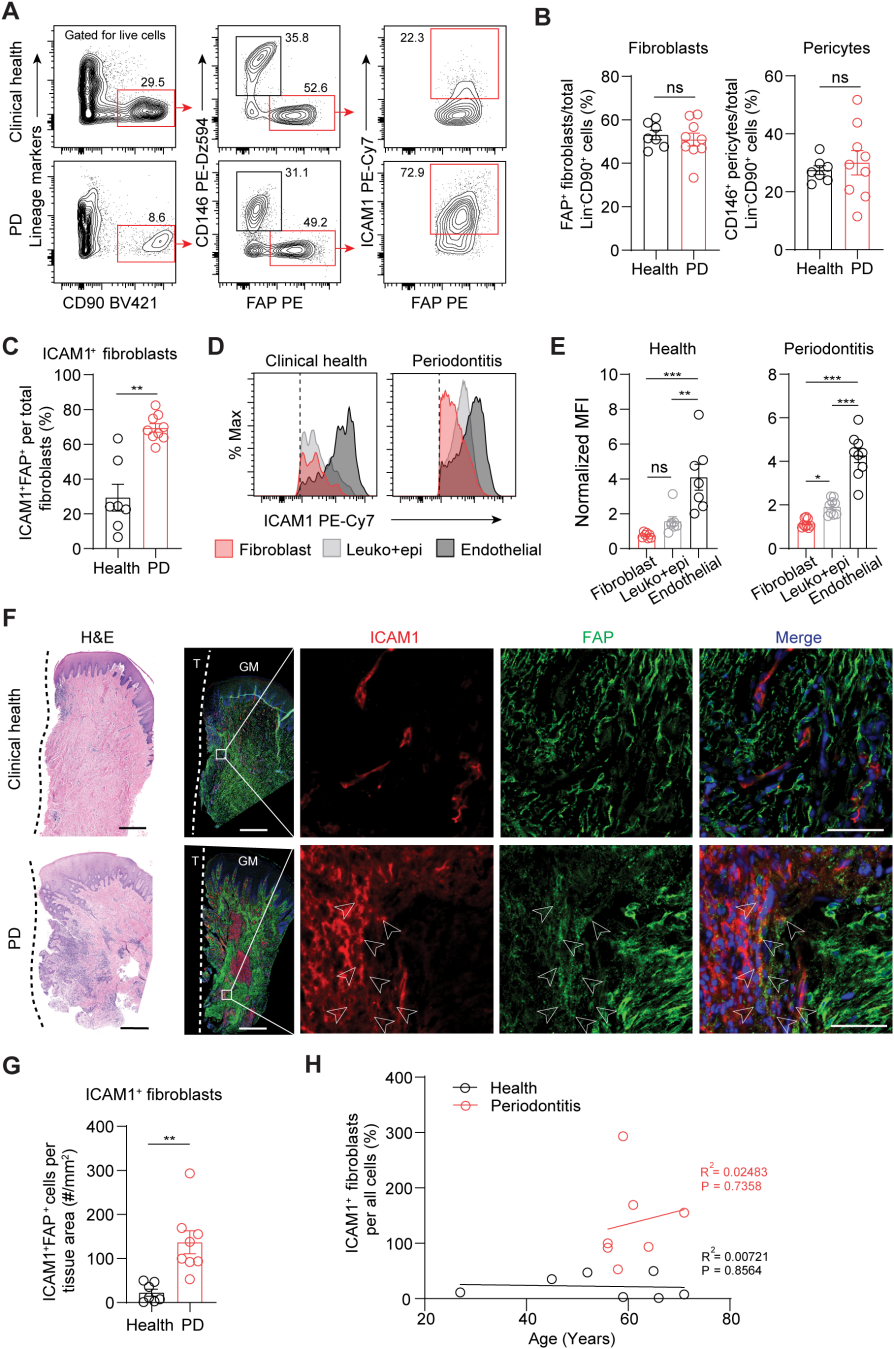
Inflammatory ICAM1^+^ fibroblasts expand in human periodontitis. **(A)** Representative flow cytometry plots of gingival tissues derived from clinical health or periodontitis (PD) patient groups. Gating strategy for quantification of ICAM1^+^ fibroblasts population (lineage^-^CD90^+^CD146^-^FAP^+^) is shown; lineage-negative selection was based on CD45 (leukocyte), CD31 (endothelial), and EpCam (epithelial) expression. **(B)** Quantification of percent fibroblasts (Lin^-^CD90^+^FAP^+^) and pericytes (Lin^-^CD90^+^CD146^+^) normalized by mesenchymal cell numbers (Lin^-^CD90^+^). **(C)** Quantification of percent ICAM1^+^ fibroblasts (Lin^-^CD90^+^FAP^+^ICAM1^+^) comparing healthy vs. PD groups. **(D)** Representative flow cytometry histogram of mean fluorescence intensity (MFI) for ICAM1 expression in fibroblast (Lin^-^FAP^+^), leukocyte+epithelial cells (CD45^+^EpCam^+^) and endothelial cells (Lin^+^CD146^+^) from healthy and PD groups. **(E)** Quantification of ICAM1 expression in fibroblast, leukocyte+epithelial cells and endothelial cells in healthy and PD groups. **(F)** Representative H&E and immunofluorescent images of healthy and PD cryosections stained with antibodies specific against ICAM1 (red) and FAP (green). Arrows point to ICAM1^+^FAP^+^ fibroblasts. Scale bar, 500μm; inset scale bar, 50μm. Dashed line demarcates the border between tooth and sulcular/junctional epithelium. **(G)** Quantification of ICAM1 fibroblast numbers normalized by tissue area (mm^2^) comparing healthy and periodontitis groups. **(H)** Scatter plot of correlation between patient age and ICAM1 fibroblast numbers in healthy and periodontitis groups. R^2^, Pearson’s correlation coefficient and, P, p-value. Each dot represents data point from individual patients (N=7-9 per group). Data represent mean ± SEM. **(B, C, G)** Student’s t-test comparing health vs. PD; **(E)** Welch’s ANOVA with Dunnett’s test for multiple comparisons; ns, not significant, *p<0.05, **p<0.01, ***p<0.001.

To validate ICAM1 as an *in vivo* surface marker for an inflammatory fibroblast subset that expands in periodontitis, we performed flow cytometry on cells prepared from freshly discarded tissues in patients with or without a periodontitis diagnosis. We utilized a fibroblast gating strategy to exclude endothelial, leukocyte, and epithelial lineage cells and used fibroblast activation protein (FAP) as a positive fibroblast marker to selectively quantify ICAM1^+^ inflammatory fibroblasts (**Figure 2A**). The overall percentage of pan-fibroblasts or pericytes normalized by lineage-negative CD90^+^ mesenchymal cell count did not differ between the clinical health and periodontitis groups (**Figure 2B**). When ICAM1^+^ fibroblasts were specifically examined, we found a significant 2.4-fold increase in the periodontitis group compared to the control group (**Figure 2C**), consistent with the patterns observed from the scRNA-seq dataset. Furthermore, we found that relative ICAM1 expression is the highest in endothelium, moderate in leukocytes, and low in fibroblasts, which aligned with our transcriptomic analysis (**Figures 1H**, **2D, E**). We further examined the spatial distribution of ICAM1^+^ fibroblasts by immunofluorescence and found increased numbers of ICAM1^+^ fibroblasts to be localized around inflammatory foci in periodontitis but not in healthy groups (**Figures 2F, G**). We considered the patient’s chronological age for this phenotype; however, it did not exhibit a positive correlation with increasing ICAM1^+^ fibroblast count (**Figure 2H**).

### Inflammatory ICAM1^+^ fibroblasts expand in a murine model of ligature-induced periodontitis

**Figure 3:**
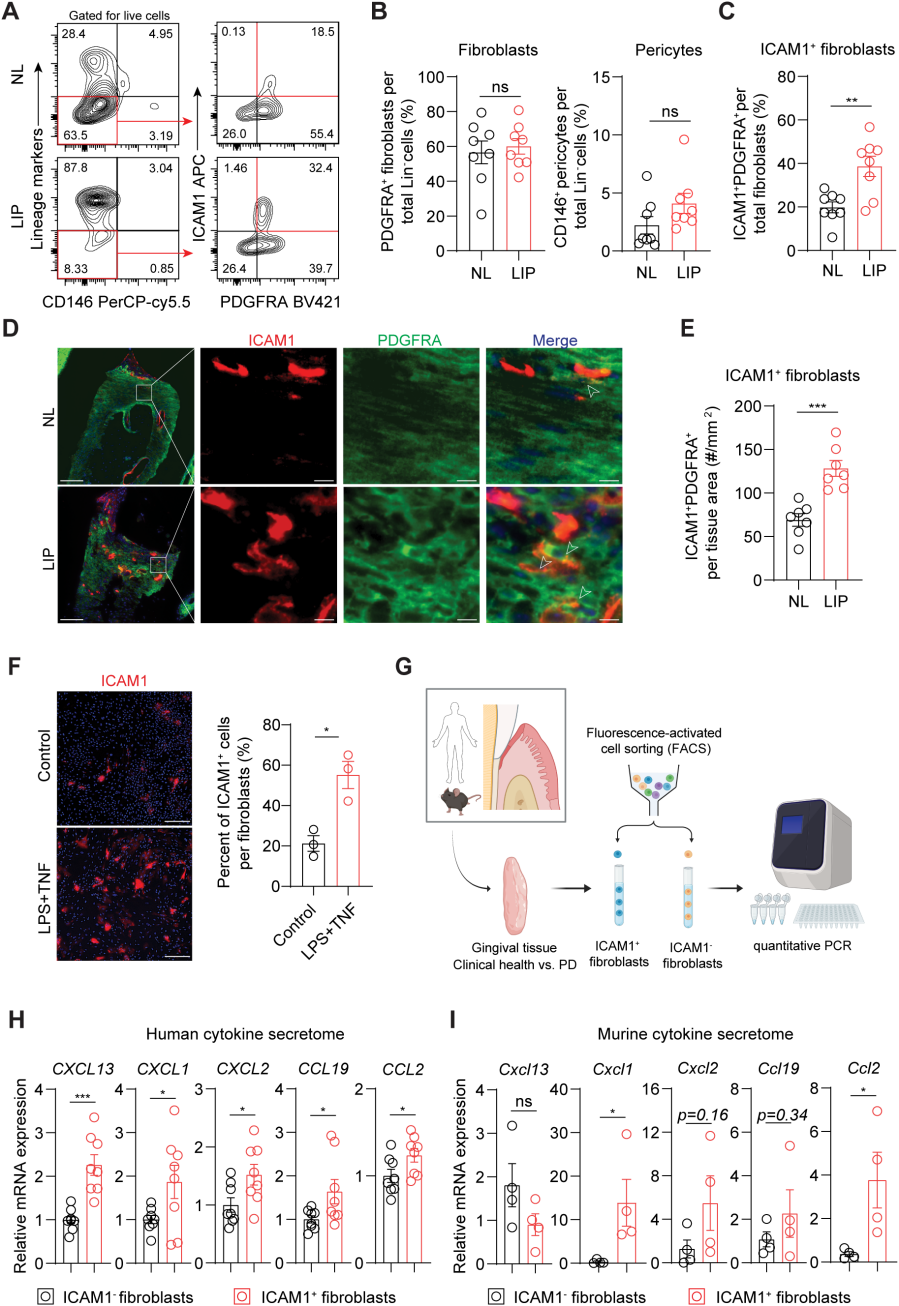
Inflammatory ICAM1^+^ fibroblasts expand in a murine model of ligature-induced periodontitis. **(A)** Flow cytometry gating strategy for analysis of lineage-negative (CD45^-^ CD31^-^ EpCam^-^ Ter119^-^) pericytes, gingival fibroblasts and ICAM1^+^ fibroblasts in non-ligated control (NL) and ligature induced periodontitis (LIP) group. **(B)** Quantification of percent fibroblasts (Lin^-^PDGFRA^+^) and pericytes (Lin^-^CD146^+^) normalized by Lin^-^ mesenchymal cell numbers in NL and LIP groups. Each dot represents one mouse as a split-mouth design. **(C)** Quantification of percent ICAM1^+^ fibroblasts (Lin^-^PDGFRA^+^ICAM1^+^) normalized by total fibroblast numbers. Each dot represents one mouse as a split-mouth design. **(D)** Representative immunofluorescent images of NL and LIP paraffin sections stained with antibodies specific against ICAM1 (red) and PDGFRA (green). Arrows point to ICAM1^+^ PDGFRA^+^ cells. Scale bar, 100μm; inset scale bar, 10μm. **(E)** Quantification of ICAM1 fibroblast numbers normalized by lamina propria area (mm^2^) comparing NL and LIP groups from the immunofluorescence experiments. **(F)** Left, representative immunocytochemistry images of primary gingival fibroblasts stained with ICAM1 antibody comparing control versus stimulated groups. Lipopolysaccharide from *P. gingivalis* (LPS, 1 ug/ml) and tumor necrosis factor alpha (TNF, 10 ng/ml) were used for stimulation. Scale bar, 20 μm. Right, quantification of ICAM1^+^ fibroblast numbers normalized by total fibroblast cells comparing control and LPS+TNF group. **(G)** Schematic diagram of qPCR for fibroblast-derived cytokines comparing FACS-sorted ICAM1^-^ and ICAM1^+^ fibroblasts in human and mouse models of periodontitis. **(H, I)** Quantification of relative mRNA expression by qPCR for CXCL13, CXCL1, CXCL2, CCL19, and CCL2 comparing sorted ICAM1^-^ and ICAM1^+^ fibroblasts. **(H)** Gingival tissue specimens from N=8 periodontitis patients; each dot represents individual patient. **(G)** Gingival tissues harvested from mice with LIP; each dot represents pooled samples from 2-3 mice for a total of 4 data points (N=10 mice). Data represent mean ± SEM. Welch’s t-test **(B–H)** and Mann-Whitney U test **(I)** comparing control vs. experimental group; *p<0.05, **p<0.01, ***p<0.001, ns, not significant.

We next used flow cytometry to test if ICAM1 expression in fibroblasts also distinguishes an expanding inflammatory fibroblast subset in murine models of ligature-induced periodontitis (LIP). Consistent with the results from human specimens, there was a significant increase in the percentage of ICAM1^+^ fibroblasts in ligated gingivae compared to non-ligated sites, without affecting the total percentage of pan-fibroblasts or pericytes when normalized to lineage-negative cell count (**Figures 3A–C**). Furthermore, immunofluorescence experiments revealed a significant increase in ICAM1^+^ fibroblastic cell numbers in the connective tissue of LIP group compared to control group (**Figures 3D, E**). Immunocytochemistry experiments on primary gingival fibroblasts demonstrated that ICAM1^+^ expression is significantly induced by adding other inflammatory and bacterial factors such as TNF and LPS (**Figure 3F**). To confirm that murine and human ICAM1^+^ fibroblasts exhibit a similar inflammatory phenotype, we sorted ICAM1^+^ and ICAM1^-^ fibroblasts from the diseased gingivae and compared mRNA expression levels of selected cytokines (**Figure 3G**) In line with the transcriptomic data, human ICAM1^+^ fibroblasts had significantly higher expression of cytokines *CXCL13, CXCL1, CXCL2, CCL19* and *CCL2* when compared to ICAM1^-^ fibroblasts (**Figure 3H**). In murine LIP models, ICAM1^+^ fibroblasts had significantly elevated expression of cytokines *Cxcl1* and *Ccl2* compared to ICAM1^-^ fibroblasts (**Figure 3I**). Despite the variable magnitudes in cytokine expression between species, these results demonstrate that ICAM1^+^ fibroblasts exhibit immune-regulatory phenotype when compared to other fibroblast populations in both humans and murine models of periodontitis.

### Perturbation of NF-κB pathway in Prx1Cre lineage exacerbates periodontal bone loss

**Figure 4:**
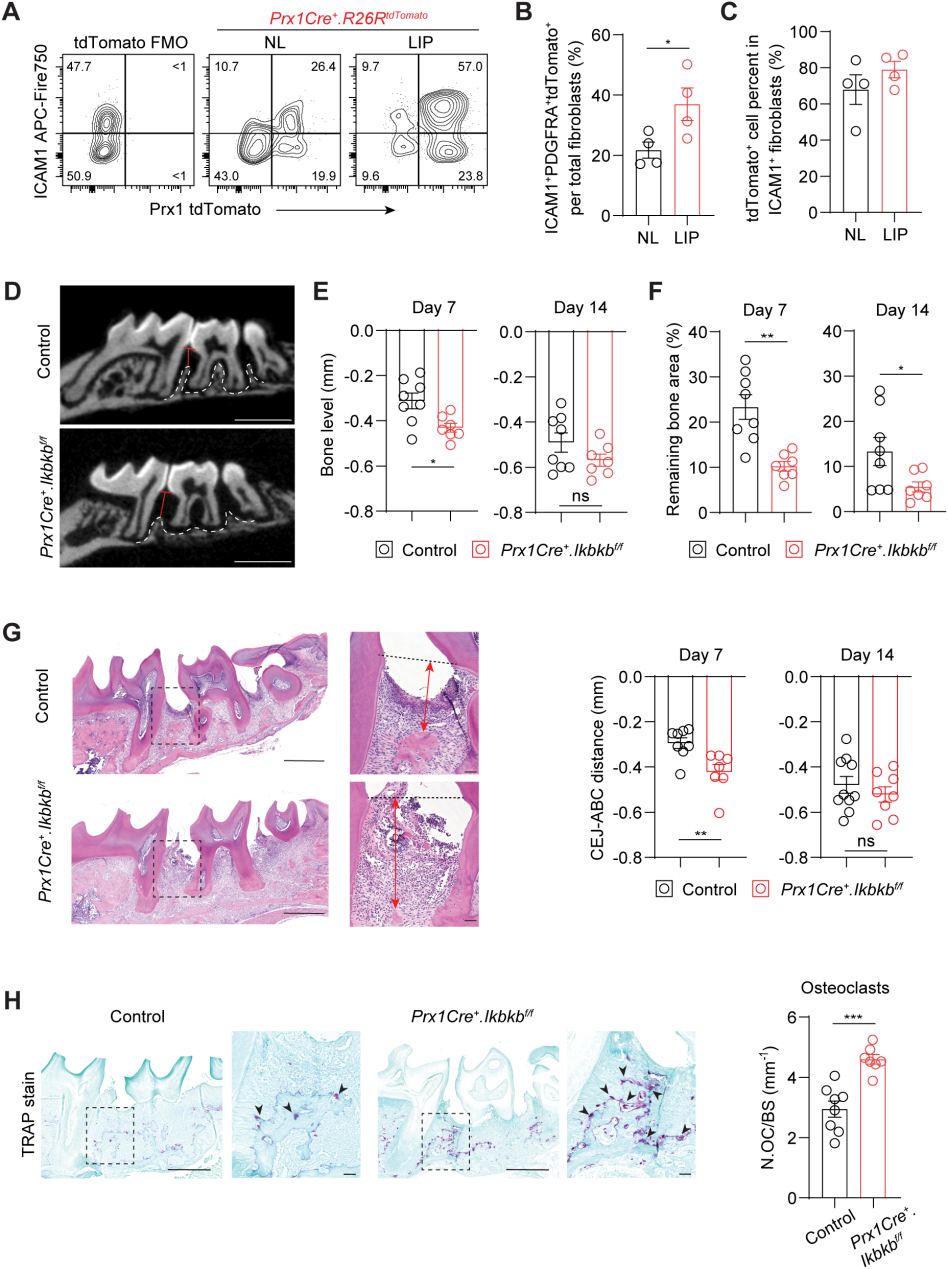
Perturbation of canonical NF-κB pathway in Prx1Cre-lineage reduces ICAM1^+^ fibroblasts and exacerbates periodontal bone loss. **(A)** Representative flow cytometry plots of ICAM1^+^ and Prx1Cre^+^ tdTomato^+^ fibroblasts in the non-ligated (NL) and ligature-induced periodontitis (LIP) groups from *Prx1Cre^+^.R26R^tdTomato^* reporter mice. Ligature was in place for 7 days (7d). FMO control from B6 mice is shown. **(B)** Quantification of percent Prx1^+^ lineage ICAM1^+^ fibroblasts (PDGFRA^+^ICAM1^+^tdTomato^+^) normalized by total fibroblast numbers comparing NL and LIP groups. **(C)** Quantification of percent Prx1^+^ lineage ICAM1^+^ fibroblasts (PDGFRA^+^ICAM1^+^tdTomato^+^) normalized by ICAM1^+^ fibroblasts (PDGFRA^+^ICAM1^+^) in NL and LIP groups. Each dot represents one mouse as a split-mouth design, N=4 mice. **(D)** Representative micro-CT images of maxillae in control and experimental (*Prx1Cre^+^.Ikbkb^f/f^*) mice that received 7d ligature. Red lines designate the distance from alveolar bone crest (ABC) to cementoenamel junction (CEJ) level, white dashed lines demarcate alveolar bone remaining. Scale bar, 1mm. **(E, F)** Quantification of bone level **(E)** and remaining bone area percent **(F)** in control and *Prx1Cre^+^.Ikbkb^f/f^* mice on 7d and 14d post-ligature. **(G)** Left, representative hematoxylin and eosin-stained images of ligated control and experimental mice 7d post-ligature. Dashed lines designate CEJ level, red arrows represent distance from CEJ to ABC. Right, quantification of CEJ-ABC distance in mm. Scale bar, 50μm. **(H)** Left, representative TRAP-stained images of ligated control and experimental mice 7d post-ligature. Arrows point to TRAP^+^ osteoclasts. Scale bar, 50μm. Right, quantification of osteoclast numbers normalized by bone lining perimeter (mm^-1^). Each dot represents data point from individual mouse (N=7-8 per group). Data represent mean ± SEM. Student’s t-test **(B, C, G, H)** or Welch’s t-test **(E, F)** comparing control vs. experimental group; ns, not significant, *p<0.05, **p<0.01, ***p<0.001.

The expansion of ICAM1^+^ fibroblasts in a diseased state may represent either a protective or detrimental inflammatory response. To determine its functional significance, we selectively targeted inflammatory oral fibroblasts by utilizing a constitutive Prx1Cre mouse model that labels immunomodulatory fibroblasts in the oral mucosa and skin (16, 25). To first confirm that Prx1-lineage fibroblasts exhibit an ICAM1^+^ phenotype and reside in tooth-associated murine gingivae, we generated *Prx1Cre^+^.R26R^tdTomato/+^* reporter mice and examined ICAM1 expression under basal and ligated conditions using flow cytometry. We quantified and found a consistently increased percentage of ICAM1^+^tdTomato^+^-lineage fibroblasts in LIP group compared to non-ligated control group (**Figures 4A, B**). Importantly, approximately 80% of ICAM1^+^ fibroblasts were tdTomato^+^ (**Figure 4C**), indicating that Prx1Cre line effectively captures most of the inflammatory ICAM1^+^ fibroblasts in the gingiva. We next investigated the impact of disrupting inflammatory functions in these fibroblasts on periodontal parameters by specifically deleting *Ikbkb* gene that encodes a kinase necessary for canonical activation of NF-κB, a master inflammatory transcription factor (26). In our integrated human scRNA-seq dataset, *PRRX1* gene was expressed in both fibroblast and pericyte clusters (**Supplementary Figure 2A**), and murine Prx1Cre model also labels pericytes in oral mucosa (16). However, *PRRX1* ^+^ fibroblasts exhibited much more elevated gene expression associated with inflammatory pathways such as TNF and NF-κB signaling relative to *PRRX1* ^+^ pericyte clusters (**Supplementary Figure 2B**), indicating that our genetic approach to perturb NF-κB pathway may have a drastic impact on Prx1Cre^+^ fibroblasts. *Ikbkb* deletion in Prx1Cre^+^ fibroblasts led to a significantly reduction in crestal bone height on day 7 and a decrease in bone area remaining on day 7 and 14 post-ligature from the µCT images (**Figures 4D–F**). The detrimental impact of *Ikbkb* deletion was confirmed by histomorphometric analysis which demonstrated significant attachment loss on day 7 LIP compared to control groups (**Figure 4G**). Furthermore, TRAP staining revealed that the number of osteoclasts along bone lining was significantly increased in the experimental group (**Figure 4H**). This effect was not a developmental defect on the alveolar bone because there were no changes to the bone level between control and *Ikbkb*-deleted groups that did not receive ligature (**Supplementary Figures 2C, D**). These results demonstrate that the inflammatory function of gingival fibroblasts is needed to prevent excessive bone loss and therefore play a protective role in the early stages of periodontal disease.

### Defective inflammatory fibroblast function is associated with deficit in macrophage recruitment and efferocytosis

**Figure 5:**
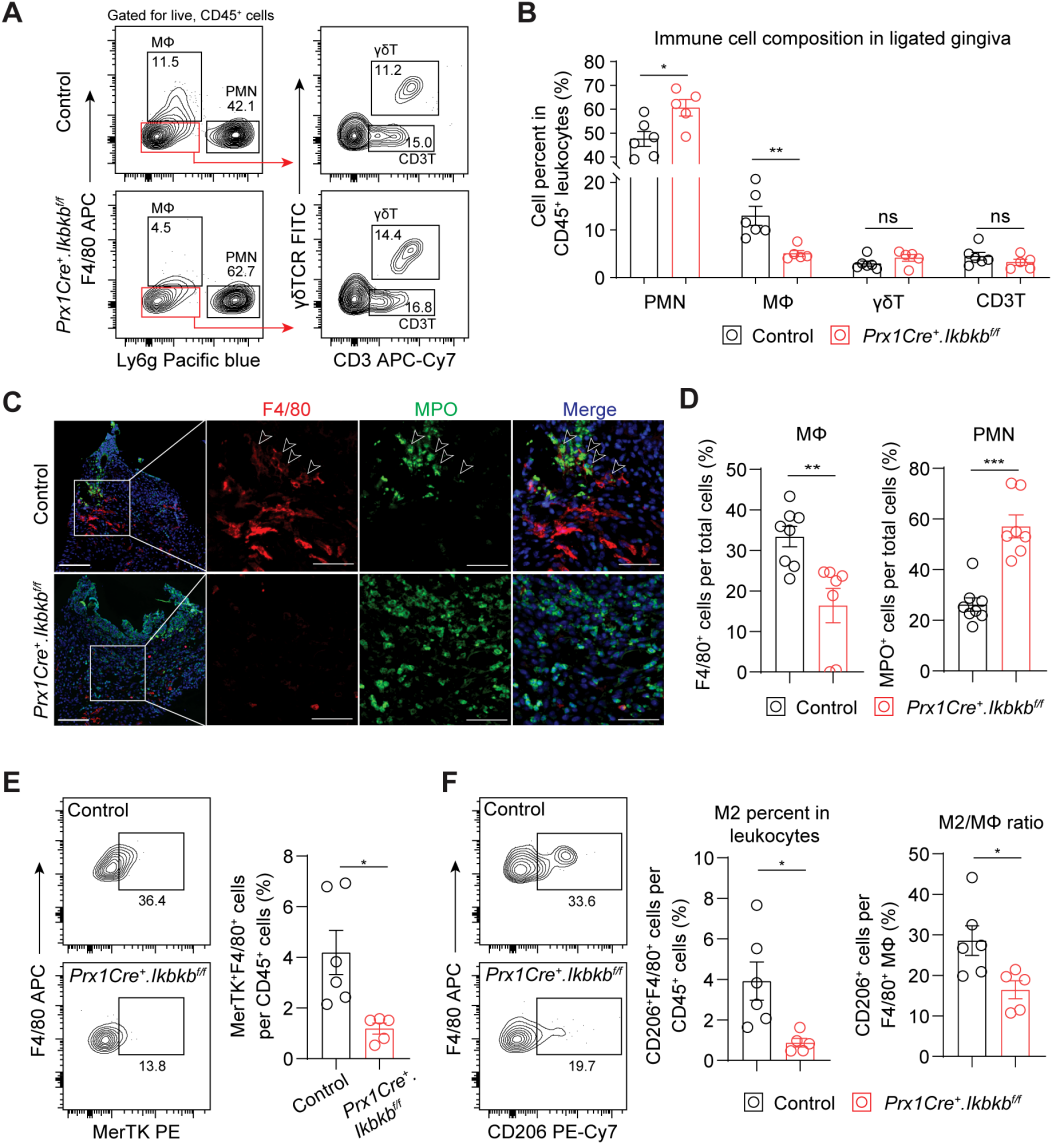
Periodontal damage by defective inflammatory fibroblasts is associated with deficit in macrophage recruitment and efferocytosis. **(A)** Representative flow cytometry plots for periodontal immunophenotyping in control and experimental (*Prx1Cre^+^.Ikbkb^f/f^*) mice on 7d post-ligature. Pre-gated population for live leukocytes (CD45^+^) is shown. **(B)** Quantification of percent neutrophils (polymorphonuclear cells, PMN; CD45^+^Ly6g^+^), macrophages (MΦ; CD45^+^F4/80^+^), CD3^+^ T cells (CD3T; CD45^+^Ly6g^-^F4/80^-^CD3^+^) and gamma delta T cells (γδT; CD45^+^Ly6g^-^F4/80^-^CD3^+^γδTCR^+^) normalized by CD45^+^ leukocytes in ligated control and experimental mice. **(C)** Representative immunofluorescent images of 7d ligated control and experimental mice in paraffin sections stained with antibodies specific against macrophage marker F4/80 (red) and neutrophil marker myeloperoxidase (MPO; green). Arrows point to F4/80 immunopositivity in proximity to MPO signal. Scale bar, 100μm; inset scale bar, 50μm. **(D)** Quantification of percent macrophages (F4/80^+^) and neutrophils (MPO^+^) normalized by total cells from immunofluorescence experiments. **(E)** Left, representative flow cytometry plots of macrophages that express MerTK (F4/80^+^ MerTK^+^), an efferocytosis marker, in 7d ligated control and experimental mice. Right, quantification of percent MerTK^+^F4/80^+^ cells normalized by CD45^+^ leukocytes. **(F)** Left, representative flow cytometry plots of M2 macrophages (F4/80^+^CD206^+^) in 7d ligated control and experimental. Right, quantification of percent M2 macrophage normalized by CD45^+^ leukocytes and by total macrophage numbers. Each dot represents data point from individual mouse (N=5-8 per group). Data represent mean ± SEM. Student’s t-test **(B, D, E)** comparing control vs. experimental group.; ns, not significant, *p<0.05, **p<0.01, ***p<0.001.

We next investigated the dysregulated inflammatory mechanism responsible for the accentuated bone loss by examining immune cell populations that contribute to periodontal pathogenesis such as neutrophils (27), macrophages (28), γδT cells (4) and CD3^+^ T cells (3). *Ikbkb* deletion in Prx1Cre lineage fibroblasts resulted in a significant 27.4% increase in Ly6g^+^ neutrophils and a remarkable 60.9% reduction in F4/80^+^ macrophages, whereas that of γδ and CD3^+^ T cells did not change (**Figures 5A, B**). Immunofluorescence on affected gingival tissues confirmed inverse patterns of macrophage and neutrophil numbers between control and experimental groups (**Figures 5C, D**). Intriguingly, F4/80 immunopositivity was intimately associated with MPO^+^ signals near the epithelium in the control group but not in the *Ikbkb* deleted group (**Figure 5C**), suggesting that a failure to clear destructive neutrophilic bodies by macrophages, termed efferocytosis (29, 30), may be affected in the experimental mice. We therefore examined the expression of MerTK, an efferocytosis marker (31, 32) and found a significant reduction in MerTK^+^F4/80^+^ macrophage numbers in the *Ikbkb* deleted group compared to control group (**Figure 5E**). Efferocytosis triggers macrophage polarization towards a pro-resolving (M2) phenotype (33, 34), thus we examined the number and percentage of M2 and found comparatively lower M2 in the experimental group compared to control (**Figure 5F**). To confirm that these observations were not simply a result of excessive neutrophil recruitment, we examined the expression of a neutrophilic cytokine CXCL1, which is highly expressed in gingival keratinocytes affected by periodontitis (35). We found that epithelium was a major source of CXCL1 and that its expression levels were similar between control and experimental groups (**Supplementary Figures 3A, B**), indicating that the intrinsic chemoattractant affinity for neutrophils may not be affected in our mouse model.

**Figure 6:**
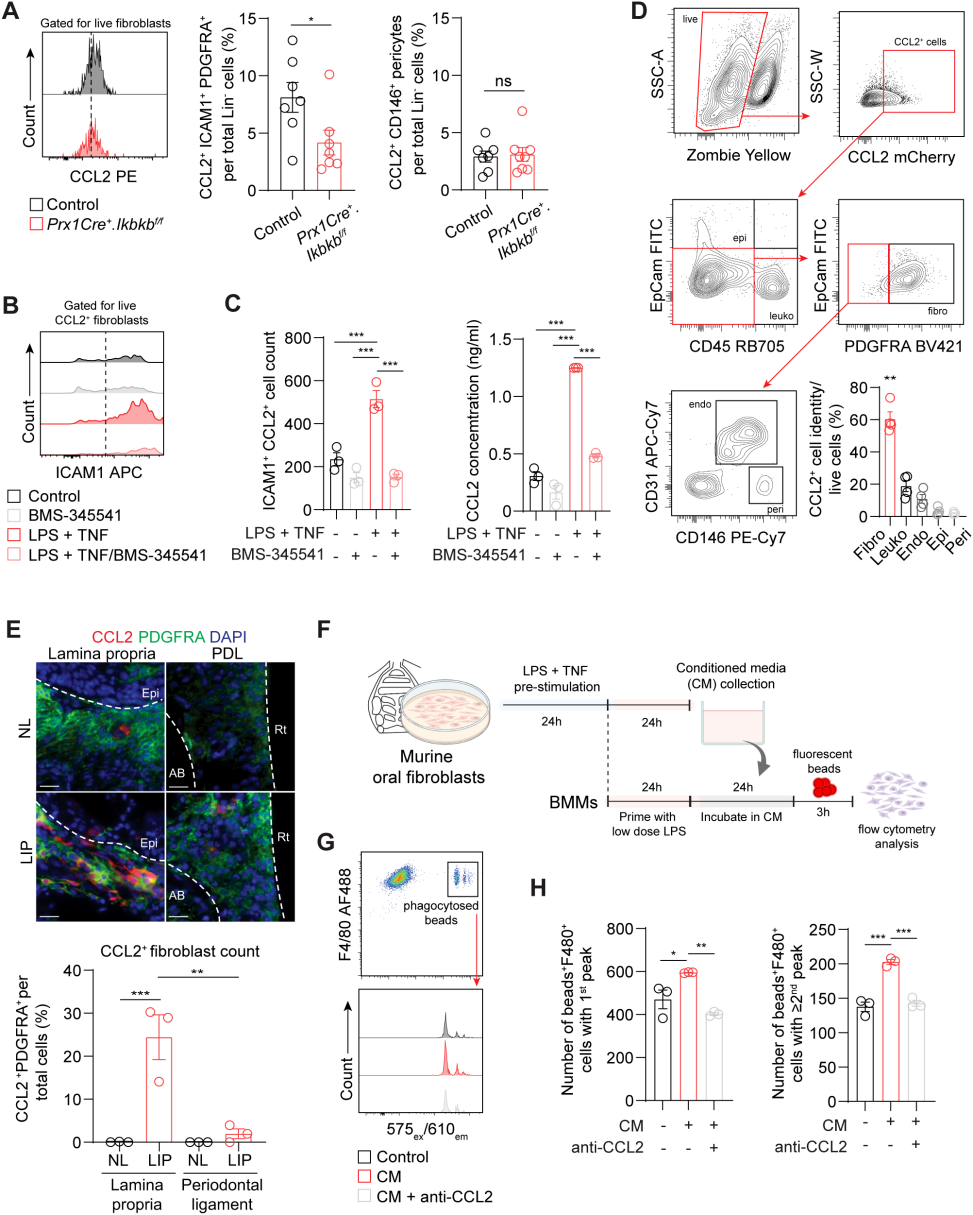
**(A)** Left, representative flow cytometry histogram of CCL2 signal in 7d ligated control and experimental mice. Middle and right, quantification of percent CCL2^+^ICAM1^+^ fibroblasts (Lin^-^PDGFRA^+^) and CCL2^+^ pericytes (Lin^-^CD146^+^) normalized by lineage-negative mesenchymal cell numbers. **(B)** Representative flow cytometry histogram of ICAM1 expression in fibroblasts pre-gated for CCL2^+^ signal in control or ICAM1^+^ oral fibroblast-enriched conditions (LPS + TNF) treated with or without BMS-345541 *in vitro.* **(C)** Left, quantification of ICAM1^+^CCL2^+^ fibroblasts cell counts by flow cytometry analysis. Right, ELISA analysis of CCL2 concentration in the supernatant of cultured control or ICAM1^+^ enriched fibroblast conditions with or without BMS-345541. **(D)** Top, representative gating strategy for CCL2^+^ cell phenotyping by flow cytometry using tissues collected from CCL2^mCherry^ reporter mice that had ligature placed for 7 days. Bottom right, quantification of CCL2^+^ fibroblasts (CD45^-^EpCAM^-^PDGFRA^+^), leukocytes (CD45^+^), endothelial cells (CD31^+^), epithelial cells (EpCAM^+^), and pericytes (CD31^-^CD146^+^) normalized by total CCL2^+^ cells. Each dot represents one mouse (N=4). **(E)** Top, representative immunofluorescent images of non-ligated control (NL) and ligature induced periodontitis (LIP) from CCL2^mCherry^ reporter mice. Paraffin sections were stained with antibodies specific against PDGFRA (green) and red fluorescent protein (red), and immunopositivity in the lamina propria and periodontal ligament space (PDL) was examined. Scale bar, 20μm.Bottom, quantification of percent CCL2^+^ fibroblasts (CCL2^+^PDFGRA^+^) normalized by total nucleated cells in field of view. N=3, split mouth design. **(F)** Schematic diagram of *in vitro* phagocytosis assay using conditioned media from ICAM1^+^ enriched oral fibroblast culture and primary bone marrow-derived macrophages. **(G)** Top, flow cytometry gating strategy for identification of double positive F4/80^+^ fluorescence beads^+^ from *in vitro* phagocytosis assay. Bottom, representative flow cytometry histogram of fluorescence beads signals showing three distinct peaks from control, conditioned media (CM), and CM + anti-CCL2 neutralization groups. **(H)** Quantification of fluorescence beads^+^ F4/80^+^ macrophage numbers per 10^4^ events. Left, number of beads^+^F4/80^+^ with a first peak (one bead) in the histogram; right, number of beads^+^F4/80^+^ with a second or third peak (two or three beads phagocytosed). N=3 each. All *in vitro* experiments were repeated independently twice. Data represents mean ± SEM. For **(A)**, one-way ANOVA test followed by pairwise t-test’s with Šidák’s correction was performed. For **(C)** Brown Forsythe ANOVA test with Dunnett’s T3 Multiple comparison test; ns, not significant, *p<0.05, **p<0.01, ***p<0.001.

To understand how inflammatory fibroblasts affect macrophage infiltration to the periodontium during induction phase of periodontitis, we examined CCL2, a monocyte and macrophage chemokine that is largely expressed by these fibroblasts (**Figure 3H**). We found that the number of ICAM1^+^CCL2^+^ fibroblasts was significantly reduced in *Prx1Cre^+^.Ikbkb^f/f^* mice (**Figure 6A**), providing key evidence for the reduced macrophage numbers in the experimental groups. In contrast, CCL2^+^ pericyte percentage remained low and unchanged by the *Ikbkb* deletion in Prx1Cre^+^ cells. *In vitro*, the enrichment of ICAM1^+^ fibroblasts with LPS and TNF pre-stimulation as in **Figure 3F** enhanced CCL2 expression by these cells and increased CCL2 concentration in the media (**Figures 6B, C**). This was effectively prevented by BMS-345541 that selectively inhibits NF-κB activity (**Figures 6B, C**), demonstrating that CCL2 expression by the ICAM1^+^ fibroblasts was dependent on the NF-κB and is consistent with the published literature in other cell types (36, 37). We next explored if fibroblasts are the major cell type that express CCL2 in our LIP model. Utilizing CCL2-mCherry reporter mice, we found that fibroblasts accounted for nearly 60% of CCL2^+^ cells, whereas leukocytes constituted approximately 20% of CCL2^+^ cells in day-7 LIP (**Figure 6D**). Immunofluorescence experiments were carried out to determine spatial distribution of the CCL2^+^ fibroblasts, and we found these cells to be localized to the lamina propria and not in the periodontal ligament space in LIP mice (**Figure 6E**). To test if the ICAM1^+^ fibroblast-derived CCL2 modulates phagocytic activity of the macrophages, we collected conditioned media from primary oral fibroblast cultures that were enriched with ICAM1^+^ as above and performed a phagocytosis assay using primary bone marrow-derived macrophages in these media (**Figure 6F**). By quantifying double-positive F4/80^+^ and internalized fluorescent bead signals, we found that macrophages phagocytosed significantly more fluorescent beads when incubated in conditioned media collected from the ICAM1^+^ fibroblast-enriched group, and this modulatory effect was diminished when neutralizing CCL2 antibody was added to the conditioned media (**Figures 6G, H**). Taken together, the results support an immune sentinel role for inflammatory ICAM1^+^ fibroblasts that modulate the recruitment and phagocytotic activity of macrophages via CCL2, safeguarding the periodontium from excessive bone loss.

## Discussion

Gingival fibroblasts have long been speculated to play an important immune sentinel role in periodontal pathogenesis (38). However, most studies rely on bulk *in vitro* culture systems that do not consider the functional and molecular heterogeneity of fibroblasts *in vivo*, thus our understanding of the role of fibroblasts in periodontitis remains incomplete. Here, we identified ICAM1 as a surface marker that selectively labels fibroblasts with inflammatory signatures in both human periodontitis and experimental mouse LIP models. Genetic perturbation of inflammatory function in fibroblasts by utilizing a Prx1Cre mouse model resulted in accelerated periodontal bone loss, supporting a protective role of these fibroblasts in periodontal disease progression. When immune cells were examined, the most notable changes were a significant reduction in macrophage numbers and an increased neutrophil count. This was attributed to insufficient efferocytosis by macrophages, which are necessary to clear neutrophilic debris (29, 30). This was linked to a reduction in CCL2^+^ fibroblast numbers in mice that had *Ikbkb* deleted. The observed immunopathology is also consistent with a recent report demonstrating the destructive role of persistent neutrophilic inflammation in experimental periodontitis (27). Our study reveals that ICAM1^+^ fibroblasts are an important initiator of inflammatory events that involve recruitment of macrophages necessary for debris clearance, thereby halting excessive periodontal bone loss.

We found that ICAM1^+^ fibroblasts expanded in diseased periodontium compared to healthy control groups in both humans and murine models. These fibroblasts shared similar inflammatory features such as elevated expression of cytokines CCL2 and CXCL1 compared to ICAM1^-^ fibroblasts. Human ICAM1^+^ had additional cytokine upregulation such as CXCL13 and CCL19 that were not present in murine ICAM1^+^ fibroblasts. We interpret this difference to be due to varying disease stage and duration found in human periodontitis that may not be present in murine LIP. In advanced human periodontitis, T and B/plasma cells predominate (39), for which CXCL13 and CCL19 expression from the ICAM1^+^ fibroblasts may be important as they are chemotactic for B and T cells, respectively (40, 41). In contrast, neutrophils and macrophages are the major immunocyte infiltration in murine experimental periodontitis (**Figure 5B**), where CCL2 expression by this fibroblast subset may be the most necessary. It is plausible that the ICAM1^+^ inflammatory fibroblasts shift their secretome profile to accommodate a transition from the innate to adaptive immune response toward the later stages of periodontal disease. This possibility is supported by the studies demonstrating plasticity of fibroblasts that acquire context-dependent inflammatory phenotype in aging (42), fibrotic disease (43), gut inflammation (6), and rheumatoid arthritis (44). Nevertheless, ICAM1^+^ fibroblasts clearly represent a specific mesenchymal subset that modulates immune responses in periodontitis, which may be useful for distinguishing inflammatory gingival fibroblasts in future studies.

Here, we utilized Prx1Cre based genetic manipulation to target fibroblast lineage cells and inhibit their immunomodulatory function by preventing NF-κB activation. ICAM1^+^ fibroblasts exhibit a transcriptional profile highly implicated with NF-κB pathways (**Figure 1G**), and inhibiting *Ikbkb*, an upstream activator of NF-κB in Prx1Cre fibroblasts exacerbated periodontal breakdown. Interestingly, ICAM1^+^ fibroblasts exhibit elevated expression of CCL2 and CXCL1 (**Figure 3H**), and these cytokines were also found to be elevated in Prx1Cre^+^ oral fibroblasts compared to lineage-negative fibroblasts (16). Given the observation that this experimental approach resulted in a drastic innate immune dysregulation, we expect potential off-target effects of *Ikbkb* deletion in other non-immunomodulatory fibroblasts to be minimal. Although Prx1Cre also labels pericytes, our scRNA-seq and pathway analysis and the results demonstrating that CCL2^+^ pericyte numbers are low and unaffected by the *Ikbkb* deletion indicate that pericytes may have less influence for the observed bone loss phenotype in our model. It is known that pericytes play an important regulatory role for limiting immune infiltration in the brain (45) and are responsive to IL-17 inflammatory pathways in rosacea (46), thus pericyte-leukocyte interaction is likely context and organ-dependent. An optimal murine model to target ICAM1^+^ inflammatory fibroblasts is not feasible, as ICAM1 is expressed in many other cell types including endothelial and immune cells (47), therefore neither a global knockout nor ICAM1 promoter driven Cre model is adequate for specifically studying ICAM1^+^ fibroblasts.

CCL2 was upregulated in both human and murine ICAM1^+^ fibroblasts, which was effectively reduced by lineage-specific deletion of *Ikbkb* thereby disrupting macrophage trafficking in the experimental periodontitis model. Our results support that CCL2-mediated immunomodulation by the fibroblasts is needed for physiologic response in periodontitis. Furthermore, they are supported by the studies demonstrating that CCL2 is essential for proper oral wound healing (24) and that exogenous application of CCL2 alleviates periodontal bone loss by modulating macrophage polarization (48). In cardiac injury, mural fibroblast-derived CCL2 is necessary for normal macrophage recruitment and positioning and thus is athero-protective (49). As ICAM1^+^ fibroblasts are a major source of CCL2, surgical approaches involving gingival transplantation enriched with these immunomodulatory fibroblasts may be a viable therapeutic approach to prevent aberrant periodontal inflammation and tissue damage.

In summary, we have identified ICAM1 as a cell surface marker to distinguish inflammatory fibroblasts that exert a protective immune function during early periodontal disease process. As the focus of our study was on characterizing the identity and role of these fibroblasts in experimental periodontitis, we did not explore the downstream events of ICAM1 activation via cell-to-cell contact mechanism with other immunocytes which is well characterized (47). ICAM1^+^ fibroblasts have been shown to directly interact and downregulate T cell responses *in vitro* (50), and similar mechanisms may affect periodontal disease progression, which may be investigated in future studies. Despite the limitations, our *in vivo* work provides strong evidence for the protective immunoregulatory role of ICAM1^+^ gingival fibroblasts in a periodontal niche. Further, it illustrates new ways to enable live selection of these cells in human and mouse models of periodontitis along with a genetic model to study them *in vivo*.

## Data Availability

The original contributions presented in the study are included in the article/**Supplementary Material**. Further inquiries can be directed to the corresponding author.

## Supplementary material

Supplementary Figure 1
**(A)** Violin plot showing number of features expressed by each sample. Dotted red lines indicate thresholds used to filter data. **(B)** Violin plot showing percentage of mitochondrial genes expressed by each sample. Dotted red lines indicate thresholds used to filter data. **(C)** Top Left, UMAP plot of unintegrated data. Top right, UMAP plot of integrated data. Bottom, UMAP plots for individual samples of integrated data. Legend applies to all plots. Samples with the AP- prefix were from GSE217720, samples labeled GM_Healthy1, GM_Healthy2, PD_Mild, and PD_Severe were from GSE164241, and all remaining samples were from GSE164241. **(D)** Juxtaposition of LISI scores calculated for unintegrated and integrated data comparing sample overlap.

Supplementary Figure 2
**(A)** UMAP plot showing *PRRX1* expression across all samples. **(B)** Left, PROGENy analysis for *PRRX1^+^* fibroblasts or pericytes in all conditions (AP, GM, and PD). Right, PROGENy analysis for *PRRX1^+^* fibroblasts or pericytes for the PD condition. **(C)** Left, representative micro-CT images of maxillae in 8 weeks old control and experimental *Prx1Cre^+^.Ikbkb^f/f^* mice. **(D)** Quantification of bone level in mm and remaining bone area between first and second molar teeth. Student’s t-test, ns, not significant. N=4-5 each group.

Supplementary Figure 3
**(A)** Left, representative H&E and immunofluorescence image of 7d ligated control and *Prx1Cre^+^.Ikbkb^f/f^* mice. Paraffin sections were stained with an antibody specific against CXCL1 and immunopositivity in the epithelium was examined. Epithelial tissue was determined via H&E staining of the same paraffin section. Scale bar, 100 μm. **(B)** Quantification of normalized mean fluorescent intensity (MFI) of CXCL1 in the epithelial area. Welch’s t-test comparing control vs. experimental groups; ns, not significant.
