# Supplementary figures and images for: ICAM1+ gingival fibroblasts modulate periodontal inflammation to mitigate bone loss
Source: Front Immunol. 2024 Nov 22;15:1484483. doi: 10.3389/fimmu.2024.1484483 (PMC11621011; doi:10.3389/fimmu.2024.1484483)

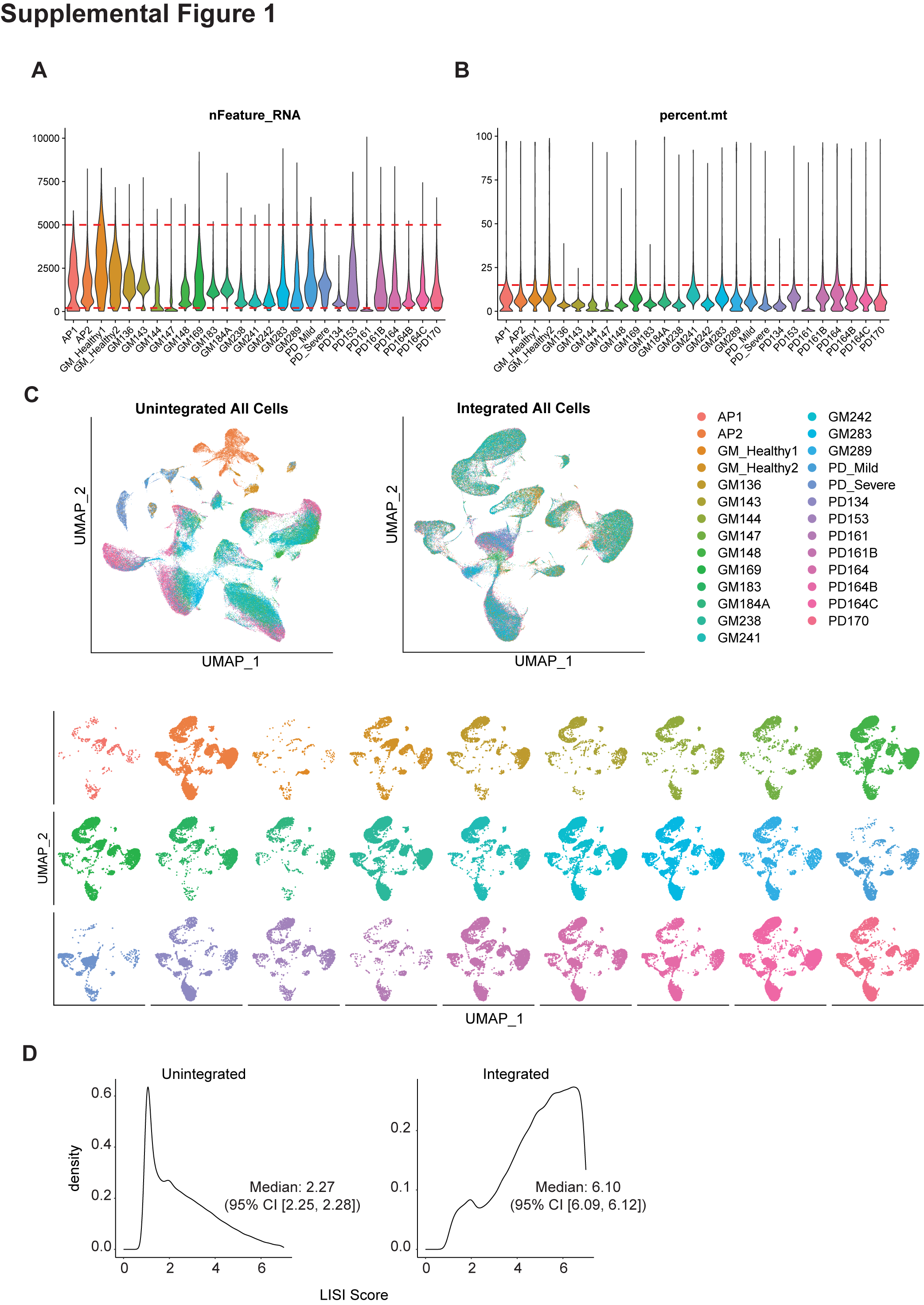

Supplement: Supplementary Figure 1 — (A) Violin plot showing number of features expressed by each sample. Dotted red lines indicate thresholds used to filter data. (B) Violin plot showing percentage of mitochondrial genes expressed by each sample. Dotted red lines indicate thresholds used to filter data. (C) Top Left, UMAP plot of unintegrated data. Top right, UMAP plot of integrated data. Bottom, UMAP plots for individual samples of integrated data. Legend applies to all plots. Samples with the AP- prefix were from GSE217720, samples labeled GM_Healthy1, GM_Healthy2, PD_Mild, and PD_Severe were from GSE164241, and all remaining samples were from GSE164241. (D) Juxtaposition of LISI scores calculated for unintegrated and integrated data comparing sample overlap. [file Image1.tif]

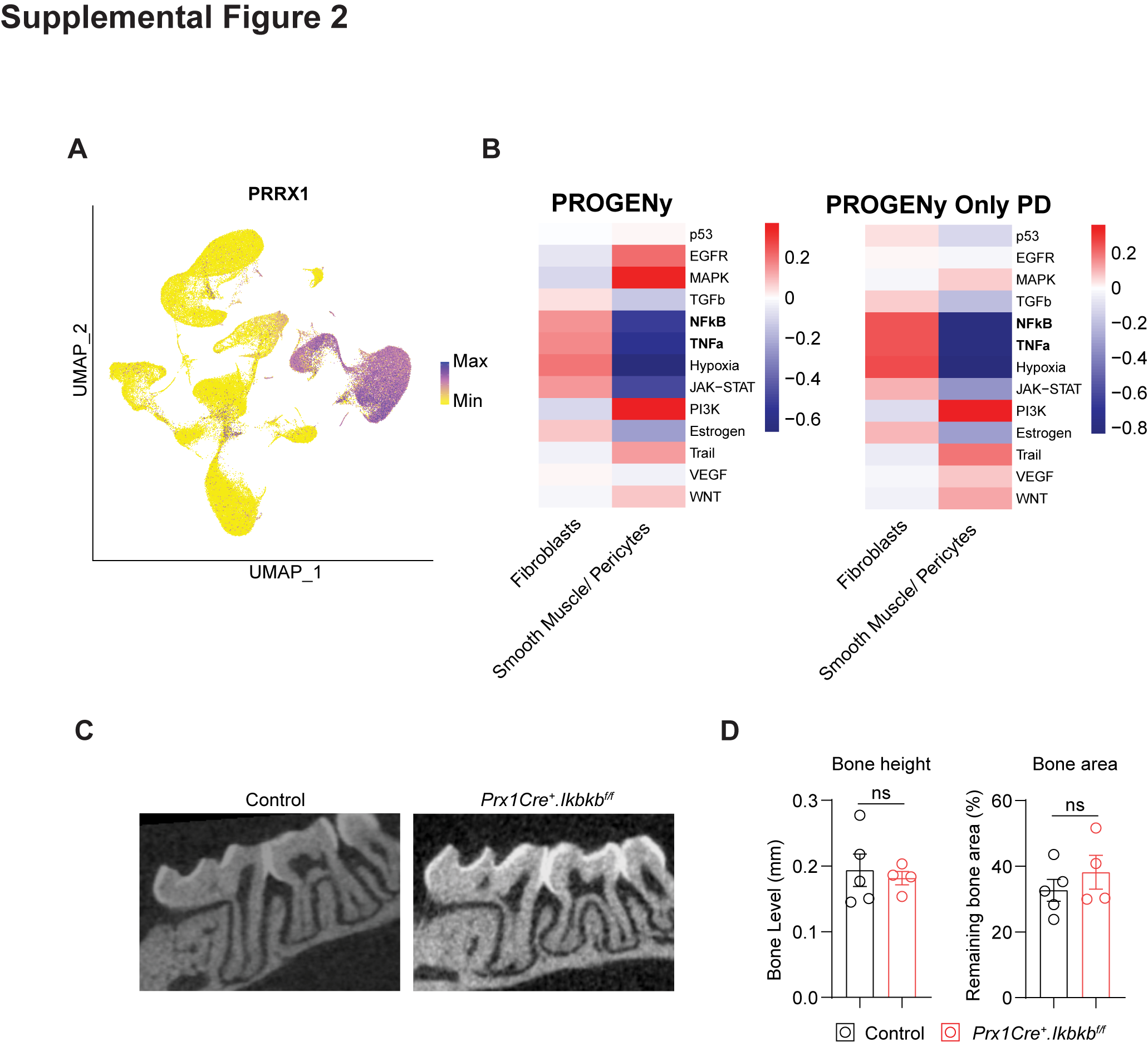

Supplement: Supplementary Figure 2 — (A) UMAP plot showing PRRX1 expression across all samples. (B) Left, PROGENy analysis for PRRX1+ fibroblasts or pericytes in all conditions (AP, GM, and PD). Right, PROGENy analysis for PRRX1+ fibroblasts or pericytes for the PD condition. (C) Left, representative micro-CT images of maxillae in 8 weeks old control and experimental Prx1Cre+.Ikbkbf/f mice. (D) Quantification of bone level in mm and remaining bone area between first and second molar teeth. Student’s t-test, ns, not significant. N=4-5 each group. [file Image2.tif]

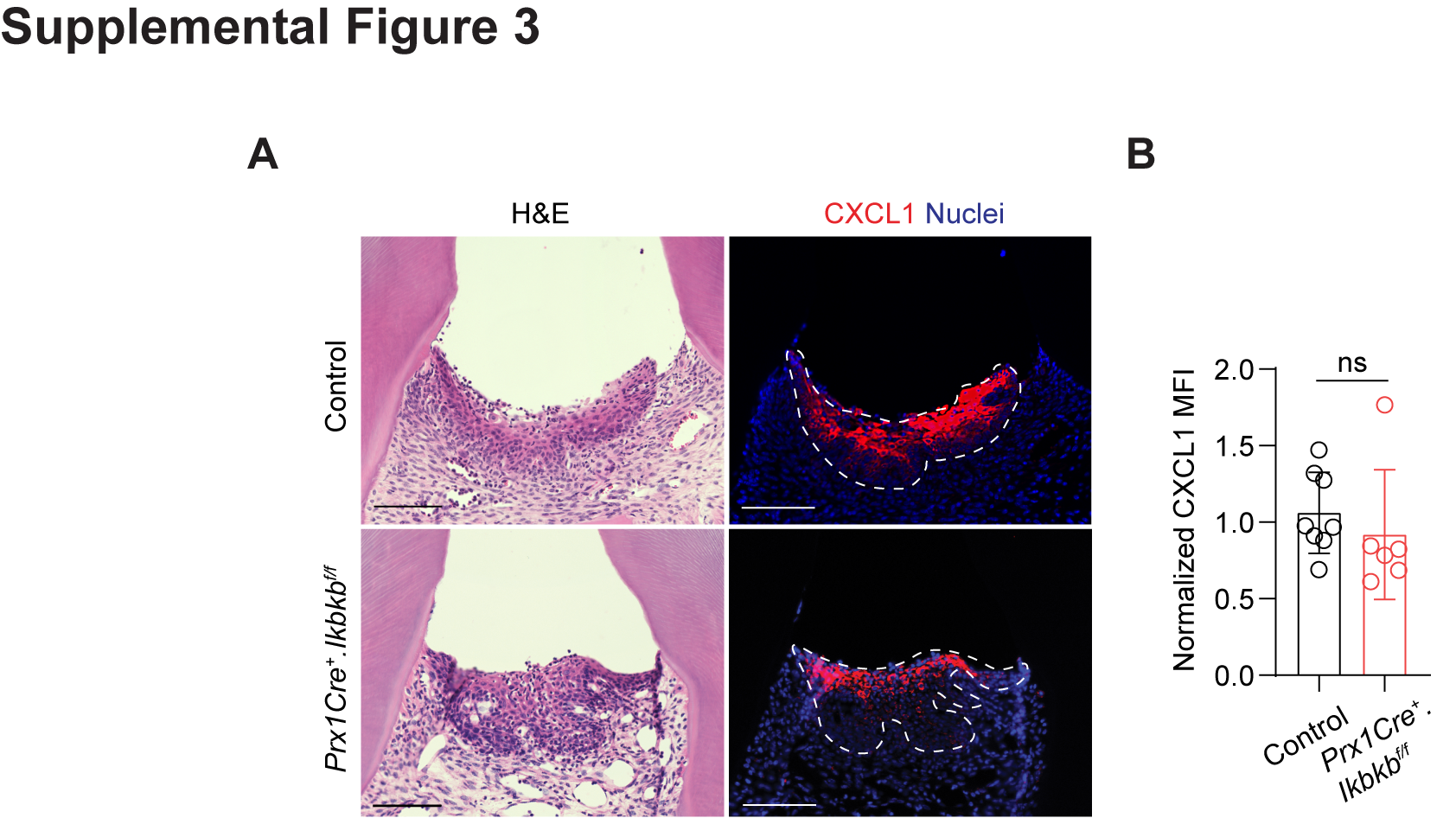

Supplement: Supplementary Figure 3 — (A) Left, representative H&E and immunofluorescence image of 7d ligated control and Prx1Cre+.Ikbkbf/f mice. Paraffin sections were stained with an antibody specific against CXCL1 and immunopositivity in the epithelium was examined. Epithelial tissue was determined via H&E staining of the same paraffin section. Scale bar, 100 μm. (B) Quantification of normalized mean fluorescent intensity (MFI) of CXCL1 in the epithelial area. Welch’s t-test comparing control vs. experimental groups; ns, not significant. [file Image3.tif]
