# Supplementary material for: ICAM1+ gingival fibroblasts modulate periodontal inflammation to mitigate bone loss
Source: Front Immunol. 2024 Nov 22;15:1484483. doi: 10.3389/fimmu.2024.1484483 (PMC11621011; doi:10.3389/fimmu.2024.1484483)
Supplement: Supplementary file 4 [file Table1.docx]

**Title:** ICAM1^+^ gingival fibroblasts modulate periodontal inflammation to mitigate bone loss

**Authors:** William S. Kim^1,†^, Kawintip Prasongyuenyong^1,2,†^, Annette Ko^1^, Rahul Debnath^1^, Zhaoxu Chen^1^, Jonathan X. Zhou^1^, Emon Shaaf^1^, Kang I. Ko^1,*^

^†^These authors contributed equally and first authorship

*Corresponding author. Email: [gank@upenn.edu](mailto:gank@upenn.edu).

| Table 1: Antibodies | | |
| --- | --- | --- |
| **Reagent or Resource** | **Source** | **Identifier (Cat. #, RRID)** |
| Sh anti FAP, polyclonal | R&D Systems | AF3715, AB_2102369 |
| Gt anti PDGFRa, polyclonal | R&D Systems | AF1062, AB_2236897 |
| GT anti MPO, polyclonal | R&D Systems | AF3667, AB_2250866 |
| Rt anti F4/80, CI:A3-1 | NOVUS | NB600-404, AB_10003219 |
| Rb anti RFP, polyclonal | Rockland | 600401379, AB_2209751 |
| Biotin mouse anti Human ICAM1/ CD54, Clone HCD54 | Biolegend | 322706, AB_535978 |
| Rt anti mouse ICAM1/ CD54, Clone YN1/1.7.4 | Biolegend | 116120, AB_10612936 |
| Dk anti Sh Cy3, polyclonal | Jackson ImmunoResearch Labs | 713165147, AB_2315778 |
| Dk anti Gt 488, polyclonal | Invitrogen | A11055, AB_2534102 |
| Dk anti Gt 594, polyclonal | Invitrogen | A11058, AB_2534105 |
| Dk anti Gt Cy3, polyclonal | Jackson ImmunoResearch Labs | 705165147, AB_2307351 |
| Dk anti Rt 647, polyclonal | Jackson ImmunoResearch Labs | 712605153, AB_2340694 |
| DK anti Rb 647, polyclonal | Jackson ImmunoResearch Labs | 711605152, AB_2492288 |
| Rat anti-mouse CD16/32, Clone 93 | Biolegend | 101302, AB_312800 |
| Rat anti Ly6g (Pacific Blue), Clone RB6-8C5 | Biolegend | 108430, AB_893556 |
| Rat anti CD45 (PE Dazzle 594), Clone 30-F11 | Biolegend | 103145, AB_2564002 |
| Rat anti MERTK (PE), Clone 2B10C42 | Biolegend | 151503, AB_2617034 |
| Rat anti CD3 (APC-Cy7), Clone 17A2 | Biolegend | 100222, AB_2242784 |
| Armenian Hamster anti gdTCR (FITC), Clone UC7-13D5 | Biolegend | 107503, AB_313312 |
| Rat anti CD206 (PE-Cy7), Clone C068C2 | Biolegend | 141720, AB_2562248 |
| Rat anti F4/80 (APC), Clone CI:A3-1 | Biolegend | MCA497APC, AB_324435 |
| Zombie Yellow | Biolegend | 77168 |
| Rat anti CD45 (FITC), Clone 30-F11 | Biolegend | 103108, AB_312973 |
| Rat anti Ter119 (FITC), Clone TER-119 | Biolegend | 116206, AB_313707 |
| Rat anti CD31 (FITC), Clone W18222B | Biolegend | 160212, AB_2910329 |
| Rat anti Epcam (FITC), Clone G8.8 | Biolegend | 118208, AB_1134107 |
| Rat anti CD54/ ICAM1 (APC), Clone YN1/1.7.4 | Biolegend | 116120, AB_10612936 |
| Rat anti CD140A/ PDGFRA (BV421), Clone APA5 | Biolegend | 135923, AB_2814036 |
| Rat anti CD146 (PerCP-Cy5.5), Clone ME-9F1 | Biolegend | 134709, AB_11204083 |
| Hamster anti CCL2 (PE), Clone 2H5 | Biolegend | 505903, AB_315409 |
| Rat anti CD140A/ PDGFRa (PE-Cy7), Clone APA5 | Biolegend | 135912, AB_2715974 |
| DAPI | Biolegend | 62248 |
| Mouse anti CD9 (APC-Fire750), Clone HI9a | Biolegend | 312113, AB_2728253 |
| Mouse anti CD90 (BV421), Clone 5E10 | Biolegend | 328121, AB_2561420 |
| Mouse anti CD146 (PE Dazzle 594), Clone P1H12 | Biolegend | 361025, AB_2783273 |
| Mouse anti FAP (PE), Clone 427819 | R&D Systems | FAB3715P, AB_3086725 |
| Mouse anti ICAM1 (PE-Cy7), Clone HA58 | Biolegend | 353115, AB_2715943 |
| Mouse anti LEPR (AF647), Clone 52263 | BD Biosciences | 564376, AB_2738777 |
| Mouse anti hCD45 (FITC), Clone 2D1 | Biolegend | 368507, AB_2566367 |
| Mouse anti hCD31 (FITC), Clone WM59 | Biolegend | 303103, AB_314329 |
| Mouse anti hEpcam (FITC), Clone 9C4 | Biolegend | 324203, AB_756077 |
| Mouse anti CD235ab (FITC), Clone HIR2 | Biolegend | 306610, AB_756046 |
| Rat anti CD54/ ICAM1 (APC-Fire750), Clone YN1/1.7.4 | Biolegend | 116126, AB_2716074 |
| Rat anti-mouse CD146 (PE-Cy7), Clone ME-9F1 | Biolegend | 134713, AB_2563108 |
| Rabbit anti CXCL1/GRO alpha /KC/CINC-1, Clone 1174A | R&D Systems | MAB4532-SP, AB_2942032 |
| Rat anti anti-mouse CD31 (APC-Cy7), Clone MEC13.3 | Biolegend | 102533, AB_2860595 |
| Rat anti CD45 (RB705), Clone 30-F11 | BD Biosciences | 570291, AB_3086726 |

**Appendix Table 1.** Antibodies used for flow cytometry and immunofluorescence experiments.

| Table 2: Primer Sequences | | | |
| --- | --- | --- | --- |
| **Gene** | **Species** | **Orientation** | **Sequence (5' to 3')** |
| CXCL13 | Mouse | Forward | GGCCACGGTATTCTGGAAGC |
| CXCL13 | Mouse | Reverse | GGGCGTAACTTGAATCCGATCTA |
| CXCL1 | Mouse | Forward | CTGGGATTCACCTCAAGAACATC |
| CXCL1 | Mouse | Reverse | CAGGGTCAAGGCAAGCCTC |
| CXCL2 | Mouse | Forward | GAAGTCATAGCCACTCTCAAGG |
| CXCL2 | Mouse | Reverse | CCTCCTTTCCAGGTCAGTTAGC |
| CCL19 | Mouse | Forward | GGGGTGCTAATGATGCGGAA |
| CCL19 | Mouse | Reverse | CCTTAGTGTGGTGAACACAACA |
| CCL2 | Mouse | Forward | TTAAAAACCTGGATCGGAACCAA |
| CCL2 | Mouse | Reverse | GCATTAGCTTCAGATTTACGGGT |
| L32 | Mouse | Forward | GAGCTGCTACAAGGCAAC |
| L32 | Mouse | Reverse | TGGACGGCTAATGCTGGT |
| CXCL13 | Human | Forward | GCTTGAGGTGTAGATGTGTCC |
| CXCL13 | Human | Reverse | CCCACGGGGCAAGATTTGAA |
| CXCL1 | Human | Forward | GAAAGCTTGCCTCAATCCTG |
| CXCL1 | Human | Reverse | CTTCCTCCTCCCTTCTGGTC |
| CXCL2 | Human | Forward | GGGCAGAAAGCTTGTCTCAA |
| CXCL2 | Human | Reverse | GCTTCCTCCTTCCTTCTGGT |
| CCL19 | Human | Forward | CTGCTGGTTCTCTGGACTTCC |
| CCL19 | Human | Reverse | AGGGATGGGTTTCTGGGTCA |
| CCL2 | Human | Forward | AGTCTCTGCCGCCCTTCT |
| CCL2 | Human | Reverse | GTGACTGGGGCATTGATTG |
| L32 | Human | Forward | GCCCAAGATCGTCAAAAAGAGA |
| L32 | Human | Reverse | GTGACTGGGGCATTGATTG |

**Appendix Table 2.** Primer sequences used for the current study.
